# (2*E*)-4-(4-Bromo­phen­yl)-2-{(2*Z*)-[1-(4-methyl­phen­yl)ethyl­idene]hydrazinyl­idene}-3-phenyl-2,3-di­hydro-1,3-thia­zole

**DOI:** 10.1107/S1600536813025506

**Published:** 2013-09-18

**Authors:** Shaaban K. Mohamed, Mehmet Akkurt, Joel T. Mague, Alaa A. Hassan, Mustafa R. Albayati

**Affiliations:** a Chemistry and Environmental Division, Manchester Metropolitan University, Manchester, M1 5GD, England; b Chemistry Department, Faculty of Science, Minia University, 61519 El-Minia, Egypt; c Department of Physics, Faculty of Sciences, Erciyes University, 38039 Kayseri, Turkey; d Department of Chemistry, Tulane University, New Orleans, LA 70118, USA; e Kirkuk University, College of Science, Department of Chemistry, Kirkuk, Iraq

## Abstract

In the title compound, C_24_H_20_BrN_3_S, the di­hydro­thia­zole ring is approximately planar, with a maximum deviation of 0.008 (2) Å, and is twisted with respect to the 4-bromo­phenyl ring, the phenyl ring and methyl­phenyl ring, making dihedral angles of 47.96 (8), 59.52 (9) and 16.96 (9)°, respectively. In the crystal, weak C—H⋯π inter­actions link inversion-related mol­ecules into supra­molecular dimers.

## Related literature

For the syntheses of similar thia­zolidine compounds, see, for example: Masoudi *et al.* (2010 ▶); Darehkordia *et al.* (2007 ▶) and for the synthesis of a related compound, see: Mohamed *et al.* (2013 ▶). For the range of biological activities of thia­zolidine-containing compounds, see: Pandeya *et al.* (1999 ▶); Shiradkar *et al.* (2007 ▶); Gududuru *et al.* (2004 ▶); Taranalli *et al.* (2009 ▶); Kumar *et al.* (2007 ▶); Rao *et al.* (2002 ▶, 2004 ▶); Barreca *et al.* (2001 ▶); Solomon *et al.* (2007 ▶); Amin *et al.* (2008 ▶); Shih & Ying (2004 ▶).
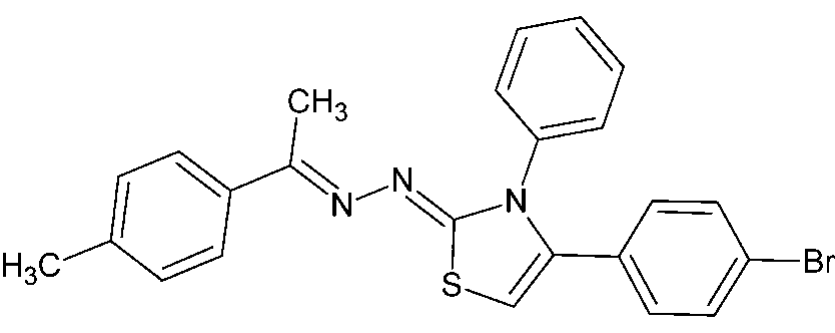


## Experimental

### 

#### Crystal data

C_24_H_20_BrN_3_S
*M* *_r_* = 462.40
Triclinic, 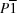

*a* = 7.9622 (5) Å
*b* = 11.2672 (7) Å
*c* = 11.6370 (7) Å
α = 96.273 (1)°
β = 94.386 (1)°
γ = 96.302 (1)°
*V* = 1027.31 (11) Å^3^
*Z* = 2
Mo *K*α radiation
μ = 2.12 mm^−1^
*T* = 150 K
0.23 × 0.17 × 0.14 mm

#### Data collection

Bruker Smart APEX CCD diffractometer
Absorption correction: multi-scan (*SADABS*; Bruker, 2013 ▶) *T*_min_ = 0.60, *T*_max_ = 0.76
18958 measured reflections
5310 independent reflections
4582 reflections with i > 2σ(i)
*R*_int_ = 0.033

#### Refinement

*R*[*F* ^2^ > 2σ(*F* ^2^)] = 0.037
*wR*(*F* ^2^) = 0.097
*S* = 1.09
5310 reflections
264 parameters
H-atom parameters constrained
Δρ_max_ = 0.97 e Å^−3^
Δρ_min_ = −0.52 e Å^−3^

### 

Data collection: *APEX2* (Bruker, 2013 ▶); cell refinement: *SAINT* (Bruker, 2013 ▶); data reduction: *SAINT*; program(s) used to solve structure: *SHELXS97* (Sheldrick, 2008 ▶); program(s) used to refine structure: *SHELXL97* (Sheldrick, 2008 ▶); molecular graphics: *ORTEP-3 for Windows* (Farrugia, 2012 ▶); software used to prepare material for publication: *WinGX* (Farrugia, 2012 ▶) and *PLATON* (Spek, 2009 ▶).

## Supplementary Material

Crystal structure: contains datablock(s) global, I. DOI: 10.1107/S1600536813025506/xu5739sup1.cif

Structure factors: contains datablock(s) I. DOI: 10.1107/S1600536813025506/xu5739Isup2.hkl

Supplementary material file. DOI: 10.1107/S1600536813025506/xu5739Isup3.cml

Additional supplementary materials:  crystallographic information; 3D view; checkCIF report

## Figures and Tables

**Table 1:** Hydrogen-bond geometry (Å, °) *Cg*4 is the centroid of the C18–C23 benzene ring.

*D*—H⋯*A*	*D*—H	H⋯*A*	*D*⋯*A*	*D*—H⋯*A*
C17—H17*A*⋯*Cg*4^i^	0.98	2.77	3.595 (3)	143

Symmetry code: (i) 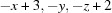.

**Fig. 1.**
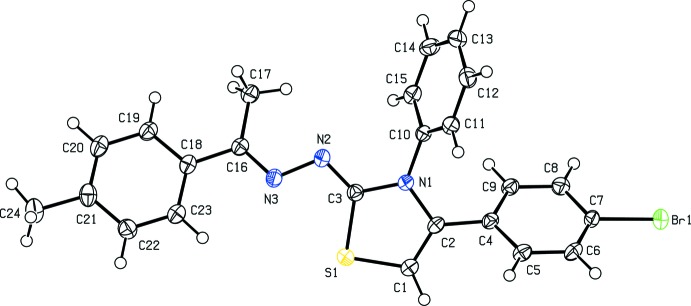
View of the title compound with 50% probability ellipsoids.

**Fig. 2.**
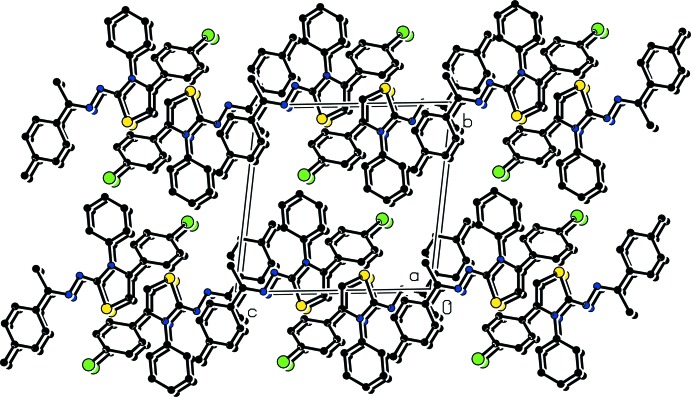
Packing of the title compound viewed down the a-axis.

## supplementary crystallographic information

### 1. Comment

The syntheses of a variety of thiazolidine-containing compounds have been reported (Masoudi *et al.*, 2010; Darehkordia *et al.*, 2007). Such compounds have been found to possess a wide range of biological properties such as antimicrobial (Pandeya *et al.*, 1999; Shiradkar *et al.*, 2007), antiproliferative (Gududuru *et al.*, 2004), anti-inflammatory, analgesic, anti-ulcer (Taranalli *et al.*, 2009; Kumar *et al.*, 2007), anti-HIV (Rao *et al.*, 2002; Rao *et al.*, 2004; Barreca *et al.*, 2001), antimalarial (Solomon *et al.*, 2007), anticonvulsant (Amin *et al.*, 2008) and antioxidant (Shih & Ying, 2004) activities. With this in mind and to further our ongoing study on the synthesis of various derivatives of thiazolidine compounds, we herein report the crystal structure of the title compound.

In Fig. 1, the thiazole ring (S1/N1/C1–C3) of the title compound is planar with an r.m.s. deviation of 0.002 Å. The 4-bromophenyl ring (C4–C9) makes a dihedral angle of 47.96 (8)° with the thiazole ring while the phenyl group (C10–C15) attached to the ring nitrogen makes a dihedral angle of 59.52 (9)°. The dihedral angle between the 4-methylphenyl ring (C18–C23) and the thiazole ring are 16.96 (9)°.

In the crystal, the packing consists of ribbons approximately parallel to [101] and assisted by C17—H17A···*Cg*4 (*Cg*4 is the centroid of the C18–C23 ring at 3 - *x*, -*y*, 2 - *z*; H17A···*Cg* = 2.77 Å, C17—H17A···*Cg* = 143°) interactions (Table 1).

### 2. Experimental

The title compound has been prepared according to our reported method (Mohamed *et al.*, 2013). The crude product has been crystallized from ethanol to afford translucent orange blocks suitable for X-ray diffraction (m.p.: 491 – 493 K).

### 3. Refinement

H atoms were positioned geometrically and allowed to ride on their parent atoms with C—H = 0.95 (aromatic H) and 0.98 Å (methyl H), with *U*_iso_(H) = 1.2*U*_iso_(C) for aromatic H atoms and *U*_iso_(H) = 1.5*U*_iso_(C) for methyl H atoms.

### Crystal data

**Table:**

C_24_H_20_BrN_3_S	*Z* = 2
*M**_r_* = 462.40	*F*(000) = 472
Triclinic, *P*1	*D*_x_ = 1.495 Mg m^−^^3^
Hall symbol: -P 1	Mo *K*α radiation, λ = 0.71073 Å
*a* = 7.9622 (5) Å	Cell parameters from 9928 reflections
*b* = 11.2672 (7) Å	θ = 2.4–29.1°
*c* = 11.6370 (7) Å	µ = 2.12 mm^−^^1^
α = 96.273 (1)°	*T* = 150 K
β = 94.386 (1)°	Block, translucent orange
γ = 96.302 (1)°	0.23 × 0.17 × 0.14 mm
*V* = 1027.31 (11) Å^3^	

### Data collection

**Table:**

Bruker Smart APEX CCD diffractometer	5310 independent reflections
Radiation source: fine-focus sealed tube	4582 reflections with i > 2σ(i)
Graphite monochromator	*R*_int_ = 0.033
Detector resolution: 8.3660 pixels mm^-1^	θ_max_ = 29.1°, θ_min_ = 1.8°
φ and ω scans	*h* = −10→10
Absorption correction: multi-scan (*SADABS*; Bruker, 2013)	*k* = −14→15
*T*_min_ = 0.60, *T*_max_ = 0.76	*l* = −15→15
18958 measured reflections	

### Refinement

**Table:**

Refinement on *F*^2^	0 restraints
Least-squares matrix: full	Hydrogen site location: inferred from neighbouring sites
*R*[*F*^2^ > 2σ(*F*^2^)] = 0.037	H-atom parameters constrained
*wR*(*F*^2^) = 0.097	W = 1/[Σ^2^(*F*O^2^) + (0.0545*P*)^2^ + 0.2353*P*] WHERE *P* = (*F*O^2^ + 2*F*C^2^)/3
*S* = 1.09	(Δ/σ)_max_ = 0.001
5310 reflections	Δρ_max_ = 0.97 e Å^−^^3^
264 parameters	Δρ_min_ = −0.52 e Å^−^^3^

### Special details

**Table:**

Experimental. The diffraction data were obtained from 3 sets of 400 frames, each of width 0.5° in ω, colllected at φ = 0.00, 90.00 and 180.00° and 2 sets of 800 frames, each of width 0.45° in φ, collected at ω = -30.00 and 210.00°. The scan time was 15 sec/frame.
Geometry. Bond distances, angles *etc*. have been calculated using the rounded fractional coordinates. All su's are estimated from the variances of the (full) variance-covariance matrix. The cell e.s.d.'s are taken into account in the estimation of distances, angles and torsion angles
Refinement. Refinement on *F*^2^ for ALL reflections except those flagged by the user for potential systematic errors. Weighted *R*-factors *wR* and all goodnesses of fit *S* are based on *F*^2^, conventional *R*-factors *R* are based on *F*, with *F* set to zero for negative *F*^2^. The observed criterion of *F*^2^ > σ(*F*^2^) is used only for calculating -*R*-factor-obs *etc*. and is not relevant to the choice of reflections for refinement. *R*-factors based on *F*^2^ are statistically about twice as large as those based on *F*, and *R*-factors based on ALL data will be even larger.

### Fractional atomic coordinates and isotropic or equivalent isotropic displacement parameters (Å^2^)

**Table:**

	*x*	*y*	*z*	*U*_iso_*/*U*_eq_	
Br1	0.30655 (3)	0.39026 (2)	0.30405 (2)	0.0337 (1)	
S1	0.89651 (6)	−0.07711 (4)	0.65607 (4)	0.0232 (1)	
N1	0.91033 (19)	0.15300 (13)	0.65666 (13)	0.0202 (4)	
N2	1.0854 (2)	0.09850 (14)	0.80890 (14)	0.0249 (5)	
N3	1.1384 (2)	−0.00294 (14)	0.85333 (14)	0.0232 (4)	
C1	0.7740 (2)	−0.01832 (17)	0.55032 (17)	0.0236 (5)	
C2	0.7931 (2)	0.10264 (17)	0.56284 (15)	0.0201 (5)	
C3	0.9789 (2)	0.06878 (16)	0.71838 (16)	0.0209 (5)	
C4	0.6892 (2)	0.17839 (16)	0.49902 (16)	0.0206 (5)	
C5	0.6570 (2)	0.15626 (18)	0.37854 (17)	0.0235 (5)	
C6	0.5459 (2)	0.22100 (18)	0.31982 (17)	0.0249 (5)	
C7	0.4664 (2)	0.30698 (17)	0.38211 (17)	0.0231 (5)	
C8	0.4987 (2)	0.33198 (17)	0.50162 (17)	0.0253 (5)	
C9	0.6109 (2)	0.26850 (17)	0.55927 (16)	0.0227 (5)	
C10	0.9755 (2)	0.27811 (16)	0.67949 (16)	0.0201 (5)	
C11	1.0510 (2)	0.33501 (17)	0.59380 (17)	0.0235 (5)	
C12	1.1144 (3)	0.45611 (18)	0.61650 (19)	0.0284 (6)	
C13	1.1018 (3)	0.51860 (18)	0.7235 (2)	0.0306 (6)	
C14	1.0260 (3)	0.46116 (19)	0.80881 (19)	0.0300 (6)	
C15	0.9628 (2)	0.34014 (18)	0.78762 (17)	0.0248 (5)	
C16	1.2045 (2)	0.01738 (17)	0.95964 (16)	0.0212 (5)	
C17	1.2183 (3)	0.13675 (18)	1.03341 (17)	0.0272 (6)	
C18	1.2755 (2)	−0.08429 (17)	1.00904 (16)	0.0217 (5)	
C19	1.3742 (2)	−0.06595 (18)	1.11581 (17)	0.0257 (5)	
C20	1.4562 (3)	−0.15780 (19)	1.15694 (18)	0.0287 (6)	
C21	1.4419 (3)	−0.27080 (19)	1.09447 (18)	0.0283 (6)	
C22	1.3378 (3)	−0.29089 (19)	0.99012 (19)	0.0287 (6)	
C23	1.2561 (2)	−0.19953 (18)	0.94795 (17)	0.0258 (5)	
C24	1.5419 (3)	−0.3684 (2)	1.1368 (2)	0.0354 (7)	
H1	0.70160	−0.06660	0.49030	0.0280*	
H5	0.71130	0.09670	0.33630	0.0280*	
H6	0.52480	0.20630	0.23770	0.0300*	
H8	0.44450	0.39190	0.54330	0.0300*	
H9	0.63510	0.28630	0.64090	0.0270*	
H11	1.05960	0.29180	0.52010	0.0280*	
H12	1.16650	0.49580	0.55810	0.0340*	
H13	1.14520	0.60130	0.73870	0.0370*	
H14	1.01710	0.50470	0.88230	0.0360*	
H15	0.91170	0.30040	0.84640	0.0300*	
H17A	1.33760	0.17120	1.04670	0.0410*	
H17B	1.17330	0.12550	1.10810	0.0410*	
H17C	1.15300	0.19140	0.99360	0.0410*	
H19	1.38520	0.01050	1.16090	0.0310*	
H20	1.52320	−0.14270	1.22930	0.0340*	
H22	1.32270	−0.36860	0.94720	0.0340*	
H23	1.18630	−0.21560	0.87680	0.0310*	
H24A	1.59110	−0.34280	1.21660	0.0530*	
H24B	1.63290	−0.38160	1.08640	0.0530*	
H24C	1.46580	−0.44330	1.13440	0.0530*	

### Atomic displacement parameters (Å^2^)

**Table:**

	*U*^11^	*U*^22^	*U*^33^	*U*^12^	*U*^13^	*U*^23^
Br1	0.0380 (1)	0.0275 (1)	0.0349 (1)	0.0071 (1)	−0.0114 (1)	0.0072 (1)
S1	0.0258 (2)	0.0195 (2)	0.0240 (2)	0.0029 (2)	0.0006 (2)	0.0024 (2)
N1	0.0203 (7)	0.0190 (7)	0.0203 (8)	0.0009 (6)	−0.0019 (6)	0.0022 (6)
N2	0.0260 (8)	0.0240 (8)	0.0239 (8)	0.0026 (6)	−0.0043 (6)	0.0046 (6)
N3	0.0222 (7)	0.0247 (8)	0.0229 (8)	0.0025 (6)	−0.0008 (6)	0.0054 (6)
C1	0.0238 (9)	0.0248 (9)	0.0209 (9)	0.0015 (7)	−0.0013 (7)	0.0008 (7)
C2	0.0186 (8)	0.0252 (9)	0.0157 (8)	0.0015 (7)	0.0008 (6)	0.0009 (6)
C3	0.0212 (8)	0.0213 (8)	0.0202 (9)	0.0020 (7)	0.0017 (7)	0.0033 (7)
C4	0.0173 (8)	0.0223 (9)	0.0210 (9)	−0.0001 (7)	−0.0010 (7)	0.0018 (7)
C5	0.0213 (8)	0.0277 (9)	0.0204 (9)	0.0025 (7)	0.0011 (7)	−0.0013 (7)
C6	0.0253 (9)	0.0310 (10)	0.0167 (9)	−0.0004 (8)	−0.0020 (7)	0.0019 (7)
C7	0.0215 (8)	0.0222 (9)	0.0247 (9)	0.0003 (7)	−0.0051 (7)	0.0057 (7)
C8	0.0255 (9)	0.0243 (9)	0.0255 (10)	0.0042 (7)	0.0005 (7)	0.0009 (7)
C9	0.0241 (9)	0.0245 (9)	0.0185 (9)	0.0025 (7)	−0.0001 (7)	0.0003 (7)
C10	0.0165 (8)	0.0200 (8)	0.0230 (9)	0.0024 (6)	−0.0017 (6)	0.0017 (7)
C11	0.0231 (9)	0.0254 (9)	0.0220 (9)	0.0044 (7)	0.0013 (7)	0.0021 (7)
C12	0.0256 (9)	0.0276 (10)	0.0330 (11)	0.0027 (8)	0.0007 (8)	0.0096 (8)
C13	0.0284 (10)	0.0216 (9)	0.0397 (12)	0.0020 (8)	−0.0064 (9)	0.0014 (8)
C14	0.0297 (10)	0.0301 (10)	0.0274 (11)	0.0055 (8)	−0.0030 (8)	−0.0067 (8)
C15	0.0224 (9)	0.0293 (10)	0.0225 (9)	0.0045 (7)	0.0004 (7)	0.0022 (7)
C16	0.0187 (8)	0.0244 (9)	0.0203 (9)	0.0004 (7)	0.0018 (7)	0.0036 (7)
C17	0.0327 (10)	0.0271 (10)	0.0213 (10)	0.0022 (8)	−0.0002 (8)	0.0033 (7)
C18	0.0184 (8)	0.0283 (9)	0.0192 (9)	0.0021 (7)	0.0030 (7)	0.0058 (7)
C19	0.0257 (9)	0.0293 (10)	0.0211 (9)	−0.0001 (7)	0.0000 (7)	0.0040 (7)
C20	0.0258 (9)	0.0373 (11)	0.0231 (10)	0.0008 (8)	−0.0007 (7)	0.0092 (8)
C21	0.0254 (9)	0.0347 (11)	0.0278 (11)	0.0058 (8)	0.0056 (8)	0.0126 (8)
C22	0.0305 (10)	0.0272 (10)	0.0292 (11)	0.0059 (8)	0.0032 (8)	0.0041 (8)
C23	0.0254 (9)	0.0298 (10)	0.0220 (9)	0.0027 (8)	0.0004 (7)	0.0035 (7)
C24	0.0367 (11)	0.0373 (12)	0.0357 (12)	0.0131 (9)	0.0028 (9)	0.0114 (9)

### Geometric parameters (Å, º)

**Table:**

Br1—C7	1.8966 (18)	C18—C23	1.397 (3)
S1—C1	1.7439 (19)	C19—C20	1.391 (3)
S1—C3	1.7573 (19)	C20—C21	1.384 (3)
N1—C2	1.406 (2)	C21—C22	1.399 (3)
N1—C3	1.385 (2)	C21—C24	1.529 (3)
N1—C10	1.437 (2)	C22—C23	1.390 (3)
N2—N3	1.398 (2)	C1—H1	0.9500
N2—C3	1.290 (2)	C5—H5	0.9500
N3—C16	1.293 (2)	C6—H6	0.9500
C1—C2	1.345 (3)	C8—H8	0.9500
C2—C4	1.471 (2)	C9—H9	0.9500
C4—C5	1.396 (3)	C11—H11	0.9500
C4—C9	1.401 (3)	C12—H12	0.9500
C5—C6	1.393 (3)	C13—H13	0.9500
C6—C7	1.384 (3)	C14—H14	0.9500
C7—C8	1.387 (3)	C15—H15	0.9500
C8—C9	1.383 (3)	C17—H17A	0.9800
C10—C11	1.383 (3)	C17—H17B	0.9800
C10—C15	1.387 (3)	C17—H17C	0.9800
C11—C12	1.391 (3)	C19—H19	0.9500
C12—C13	1.378 (3)	C20—H20	0.9500
C13—C14	1.384 (3)	C22—H22	0.9500
C14—C15	1.389 (3)	C23—H23	0.9500
C16—C17	1.503 (3)	C24—H24A	0.9800
C16—C18	1.483 (3)	C24—H24B	0.9800
C18—C19	1.401 (3)	C24—H24C	0.9800
			
C1—S1—C3	90.57 (9)	C21—C22—C23	121.4 (2)
C2—N1—C3	113.95 (15)	C18—C23—C22	120.76 (18)
C2—N1—C10	124.72 (15)	S1—C1—H1	124.00
C3—N1—C10	120.74 (15)	C2—C1—H1	124.00
N3—N2—C3	111.32 (15)	C4—C5—H5	120.00
N2—N3—C16	114.04 (16)	C6—C5—H5	120.00
S1—C1—C2	112.77 (14)	C5—C6—H6	120.00
N1—C2—C1	112.72 (16)	C7—C6—H6	120.00
N1—C2—C4	121.09 (16)	C7—C8—H8	120.00
C1—C2—C4	125.66 (16)	C9—C8—H8	120.00
S1—C3—N1	109.97 (12)	C4—C9—H9	120.00
S1—C3—N2	127.42 (14)	C8—C9—H9	119.00
N1—C3—N2	122.60 (16)	C10—C11—H11	120.00
C2—C4—C5	120.73 (16)	C12—C11—H11	120.00
C2—C4—C9	120.26 (16)	C11—C12—H12	120.00
C5—C4—C9	118.85 (16)	C13—C12—H12	120.00
C4—C5—C6	120.42 (17)	C12—C13—H13	120.00
C5—C6—C7	119.41 (18)	C14—C13—H13	120.00
Br1—C7—C6	119.81 (14)	C13—C14—H14	120.00
Br1—C7—C8	118.99 (13)	C15—C14—H14	120.00
C6—C7—C8	121.18 (17)	C10—C15—H15	120.00
C7—C8—C9	119.13 (17)	C14—C15—H15	120.00
C4—C9—C8	120.97 (17)	C16—C17—H17A	109.00
N1—C10—C11	119.67 (16)	C16—C17—H17B	109.00
N1—C10—C15	119.49 (16)	C16—C17—H17C	109.00
C11—C10—C15	120.85 (17)	H17A—C17—H17B	109.00
C10—C11—C12	119.43 (18)	H17A—C17—H17C	109.00
C11—C12—C13	120.2 (2)	H17B—C17—H17C	109.00
C12—C13—C14	120.13 (19)	C18—C19—H19	119.00
C13—C14—C15	120.4 (2)	C20—C19—H19	119.00
C10—C15—C14	119.06 (18)	C19—C20—H20	119.00
N3—C16—C17	124.44 (17)	C21—C20—H20	119.00
N3—C16—C18	116.73 (17)	C21—C22—H22	119.00
C17—C16—C18	118.80 (16)	C23—C22—H22	119.00
C16—C18—C19	120.75 (17)	C18—C23—H23	120.00
C16—C18—C23	121.50 (16)	C22—C23—H23	120.00
C19—C18—C23	117.62 (17)	C21—C24—H24A	109.00
C18—C19—C20	121.12 (18)	C21—C24—H24B	109.00
C19—C20—C21	121.2 (2)	C21—C24—H24C	109.00
C20—C21—C22	117.8 (2)	H24A—C24—H24B	109.00
C20—C21—C24	120.7 (2)	H24A—C24—H24C	110.00
C22—C21—C24	121.52 (19)	H24B—C24—H24C	109.00
			
C1—S1—C3—N2	178.97 (17)	C5—C4—C9—C8	2.3 (3)
C3—S1—C1—C2	−0.78 (14)	C4—C5—C6—C7	−0.5 (3)
C1—S1—C3—N1	−0.03 (14)	C5—C6—C7—Br1	−176.96 (14)
C3—N1—C10—C11	116.25 (19)	C5—C6—C7—C8	1.7 (3)
C3—N1—C2—C1	−1.4 (2)	C6—C7—C8—C9	−0.8 (3)
C3—N1—C10—C15	−63.3 (2)	Br1—C7—C8—C9	177.83 (13)
C10—N1—C3—N2	10.2 (3)	C7—C8—C9—C4	−1.2 (3)
C10—N1—C2—C4	−18.1 (2)	N1—C10—C11—C12	−179.80 (17)
C10—N1—C3—S1	−170.78 (13)	C11—C10—C15—C14	0.5 (3)
C2—N1—C3—N2	−178.25 (16)	C15—C10—C11—C12	−0.2 (3)
C3—N1—C2—C4	170.66 (15)	N1—C10—C15—C14	−179.91 (18)
C2—N1—C10—C15	126.02 (18)	C10—C11—C12—C13	0.0 (3)
C10—N1—C2—C1	169.77 (16)	C11—C12—C13—C14	0.0 (4)
C2—N1—C10—C11	−54.4 (2)	C12—C13—C14—C15	0.3 (4)
C2—N1—C3—S1	0.81 (18)	C13—C14—C15—C10	−0.6 (3)
N3—N2—C3—S1	2.6 (2)	N3—C16—C18—C19	169.30 (16)
C3—N2—N3—C16	−161.26 (16)	N3—C16—C18—C23	−6.5 (2)
N3—N2—C3—N1	−178.52 (15)	C17—C16—C18—C19	−8.8 (2)
N2—N3—C16—C17	2.6 (3)	C17—C16—C18—C23	175.40 (17)
N2—N3—C16—C18	−175.39 (14)	C16—C18—C19—C20	−173.07 (18)
S1—C1—C2—N1	1.38 (19)	C23—C18—C19—C20	2.9 (3)
S1—C1—C2—C4	−170.28 (14)	C16—C18—C23—C22	173.41 (18)
N1—C2—C4—C5	140.44 (17)	C19—C18—C23—C22	−2.5 (3)
C1—C2—C4—C9	126.8 (2)	C18—C19—C20—C21	−0.6 (3)
C1—C2—C4—C5	−48.6 (3)	C19—C20—C21—C22	−2.0 (3)
N1—C2—C4—C9	−44.2 (2)	C19—C20—C21—C24	176.0 (2)
C2—C4—C9—C8	−173.15 (16)	C20—C21—C22—C23	2.3 (3)
C2—C4—C5—C6	174.01 (17)	C24—C21—C22—C23	−175.6 (2)
C9—C4—C5—C6	−1.4 (3)	C21—C22—C23—C18	−0.1 (3)

### Hydrogen-bond geometry (Å, º)

Cg4 is the centroid of the C18–C23 benzene ring.

**Table:**

*D*—H···*A*	*D*—H	H···*A*	*D*···*A*	*D*—H···*A*
C17—H17*C*···N2	0.98	2.28	2.713 (3)	105
C17—H17*A*···*Cg*4^i^	0.98	2.77	3.595 (3)	143

Symmetry code: (i) −*x*+3, −*y*, −*z*+2.
